# Effects of Saponins on Lipid Metabolism: A Review of Potential Health Benefits in the Treatment of Obesity

**DOI:** 10.3390/molecules21101404

**Published:** 2016-10-20

**Authors:** Mariangela Marrelli, Filomena Conforti, Fabrizio Araniti, Giancarlo A. Statti

**Affiliations:** 1 Department of Pharmacy, Health and Nutritional Sciences, University of Calabria, Rende (CS) I-87036, Italy; filomena.conforti@unical.it (F.C.); g.statti@unical.it (G.A.S.); 2 Department of AGRARIA, University “Mediterranea” of Reggio Calabria, Reggio Calabria (RC) I-89124, Italy; fabrizio.araniti@unirc.it

**Keywords:** lipid metabolism, medicinal plant, obesity, phytochemicals, saponins

## Abstract

Obesity is one of the greatest public health problems. This complex condition has reached epidemic proportions in many parts of the world, and it constitutes a risk factor for several chronic disorders, such as hypertension, cardiovascular diseases and type 2 diabetes. In the last few decades, several studies dealt with the potential effects of natural products as new safe and effective tools for body weight control. Saponins are naturally-occurring surface-active glycosides, mainly produced by plants, whose structure consists of a sugar moiety linked to a hydrophobic aglycone (a steroid or a triterpene). Many pharmacological properties have been reported for these compounds, such as anti-inflammatory, immunostimulant, hypocholesterolemic, hypoglycemic, antifungal and cytotoxic activities. The aim of this review is to provide an overview of recent studies about the anti-obesity therapeutic potential of saponins isolated from medicinal plants. Results on the in vitro and in vivo activity of this class of phytochemicals are here presented and discussed. The most interesting findings about their possible mechanism of action and their potential health benefits in the treatment of obesity are reported, as well.

## 1. Introduction

Obesity is a serious and increasing health problem consisting of an excessive growth of adipose tissue. Introduced in the International Classification of Diseases (ICD) only in the 1950s, it is now reaching epidemic proportions worldwide, tripling its prevalence since the 1980s in many European countries and affecting a large percentage of the population [1]. The prevalence of overweight and obese people is extremely high in parts of Europe, the U.S. and Mexico. However, a rising obesity incidence was observed also in regions such as South America and Asia, where the incidence is still low [2]. Referring to this global phenomenon, a new word, “globesity”, has been coined [3].

The rise of obesity has been attributed to different potential factors, genetic background, diet and physical activity being the major ones. A genetic predisposition to obesity has been recognized to affect the energy balance equation resulting from energy input and output. However, metabolic factors, high-fat diets and sedentary lifestyle are considered important causes in obesity etiology [4,5].

This severe health problem is associated with an increased risk of several diseases, including type II diabetes, cardiovascular diseases, cancer and osteoarthritis, as well as asthma and chronic back pain. Therefore, the prevention and the treatment of obesity is extremely important [6].

Nowadays, the treatment of obesity consists of a reduction of caloric dietary intake combined with an increase in physical activity. A pharmacologic treatment is preferred when the behavioral approach is not enough to obtain weight control. Different drugs have been marketed in the last few years, which have been successively withdrawn because of their serious adverse effects. Dinitrophenol was the first drug used for weight loss. It was introduced in the 1930s for the treatment of obesity, but rapidly substituted by amphetamines, chosen for their ability to suppress appetite. Furthermore, these last molecules were banned by the Food and Drug Administration (FDA) because of their severe side effects. Among other kinds of drugs whose use was attempted (e.g., phentermine and fenfluramine), sibutramine was one of the most important. This norepinephrine and serotonin reuptake inhibitor was approved for the treatment of obesity in 1997 by the FDA and in 1999 in the European Union. This drug is able to affect both food intake and energy expenditure. However, also sibutramine was withdrawn from European, U.S. and Canadian markets, because of cardiovascular concerns. The cannabinoid receptor antagonist rimonabant, which acts in the nervous system by blocking cannabinoid type 1 (CB1) receptors involved in the control of food intake, followed a similar trend of its predecessors. In fact, its sale was approved in Europe in 2006 and suspended due to the risk of psychiatric disorders [7]. Actually, orlistat, a semisynthetic hydrogenated derivative of the natural lipase inhibitor produced by *Streptomyces toxytricini* [8], is the only commonly-used anti-obesity drug. This compound is the only anti-obesity medication currently approved by the European Agency for the Evaluation of Medicinal Products (EMEA) in Europe for long-term use, while in the USA, besides orlistat, phentermine is also available, even if only for short-term use [7].

Approved in 1998, orlistat is a potent gastrointestinal lipase inhibitor able to prevent dietary fat absorption by 30%, inhibiting both pancreatic and gastric lipase. However, orlistat might not be well tolerated since side effects, such as diarrhea, fecal incontinence, flatulence, bloating and dyspepsia, are commonly developed [9].

Because of the adverse effects associated with this available lipid-lowering agent, there is a growing interest in herbal remedies, aiming to find well-tolerated naturally-effective drugs [10].

Different natural compounds are able to modulate obesity through various mechanisms of action. In the last decade, a high number of reviews has been focused on the potential use of natural products in the treatment of obesity, and different classes of phytochemicals have been explored and reviewed [11,12,13,14,15,16,17,18,19,20,21,22,23].

For instance, the potential role of polyphenols has been largely investigated and recently reviewed. Commonly-consumed phenols, such as green tea catechins, curcumin and resveratrol, are able to reduce adipocytes viability, suppress adipocyte differentiation and triglyceride accumulation and stimulate lipolysis [24,25,26,27].

Among the biologically-active classes of phytochemicals, several saponins have been demonstrated to lower body weight and serum lipid levels. In the present manuscript, the most interesting findings about the in vitro and in vivo anti-obesity activity of this class of phytochemicals and saponins containing plant extracts, their mechanisms of action and their potential health benefits in the treatment of obesity are reviewed. The literature search was conducted using various electronic databases, mainly PubMed and Google Scholar. More than forty interesting manuscripts dealing with the anti-obesity activity of saponins or saponin-containing extracts were found, together with various publications about the anti-obesity potential of plant extracts.

## 2. Obesity and Plant Secondary Metabolites

Plant natural products are a priceless source of medicinal compounds [28,29], fibers [30], flavorings [31], fragrances [32,33], natural herbicides [34,35,36,37] and pesticides [38,39].

According to the WHO, medicinal plants are important tools for healthcare in developing countries. Nowadays, since synthetic anti-obesity drugs are characterized by important side effects, we are currently assisting to increase the scientific interest towards natural products. Moreover, the “omics” technologies (genomics, proteomics, transcriptomics and metabolomics) allow one to validate the use of traditional medicines easily and to identify new natural compounds and their mechanism of action [13].

Different classes of phytochemicals have been shown to modulate body weight. It has been demonstrated that polyphenols, such as catechins and anthocyanins, modulate molecular pathways involved in energy metabolism [25].

The anti-obesity properties of polyphenols may be due to their ability to interact with preadipocytes, adipose stem cells and immune cells of the adipose tissues [24].

Some clinical and epidemiological studies have suggested the beneficial effects of the consumption of green tea, rich in catechins, for its anti-obesity properties. Green tea catechins, above all epigallocatechin gallate (EGCG), are able to inhibit fat absorption and suppress adipocyte differentiation and proliferation [25].

Resveratrol (3,4′,5-trihydroxystilbene), contained in grapes and red wine, is another example of a phenolic compound with anti-obesity potential. This naturally-occurring polyphenolic compound is able to inhibit preadipocyte differentiation, decrease adipocyte proliferation and lipogenesis and promote lipolysis [24].

Furthermore, some terpenes have been pointed out for potential effects in body weight control [11,40]. The diterpene carnosic acid, isolated from the leaves of *Salvia officinalis* L., is a pancreatic lipase (PL) inhibitor and has been demonstrated to suppress fat weight increase in high-fat diet-fed mice [41]. Phytosterols [42] and some alkaloids [23,40] are other plant secondary metabolites that appear to be important in body weight control. Interestingly, also soy proteins’ consumption has been suggested to have efficacy against obesity [43].

According to these findings, a minimum daily intake of 400 g of fruits and vegetables is recommended for the prevention of obesity and other diseases, such as cancer and heart problems [18].

Some natural anti-obesity agents from medicinal plants have reached clinical trials. However, thousands of plants, some of which are traditionally used against obesity, have not been investigated yet. Thus, potentially, new effective molecules could be discovered, and further research in this field is needed to deeply investigate herbal anti-obesity products, their mechanisms of action and, above all, to verify the lack of toxicity and side effects [13].

The potential anti-obesity activity of different saponins has been also highlighted in the last few decades, and the activity of some of these secondary metabolites has been reported in previous reviews about the therapeutic anti-obesity potential of natural compounds [11,23,40].

The purpose of the present review is to specifically focus attention on this class of compounds, summarizing all obtained results and actual knowledge. Different saponins and active plant extracts are here grouped based on different mechanisms of action, such as lipase inhibition, suppression of appetite signals or adipogenesis regulation.

## 3. Saponins: Structure and Medicinal Properties

Saponins are naturally-occurring surface-active glycosides mainly produced by plants, besides some bacteria and lower marine animals. Their structure consists of a sugar moiety linked to a hydrophobic aglycone called sapogenin. The sugar moiety may contain glucose, galactose, rhamnose, methylpentose, glucuronic acid or xylose, while the aglycone portion may be a steroid or a triterpene [44]. Steroidal saponins are mainly abundant in monocotyledons, while dicotyledons predominantly contain triterpenoid saponins [45]. These phytochemicals are so called because of their ability to form stable soap-like foams in aqueous solutions [44]. Thanks to this property, saponins are used as natural surfactants in cleansing products for personal care, such as foam baths, shower gels, liquid soaps, shampoos and toothpastes [45].

Saponins can be toxic if given intravenously [46]. These compounds are known for their hemolytic activity on human erythrocytes, which depends on the type of aglycone and sugar chains. This property is due to the interaction with sterols present in the erythrocyte membrane, which lead to an increase of membrane permeability and the consequent loss of hemoglobin [47]. These molecules can also act as fish poison [48], and some saponin-containing plants are toxic for ruminants, leading to gastroenteritis, diarrhea and even liver and kidney degeneration [49].

Besides these effects, many pharmacological properties, such as antifungal, insecticidal, anthelmintic, cytotoxic, anti-inflammatory, immunostimulant, hypocholesterolemic and hypoglycemic, have been ascribed to these compounds [44,45,50].

The cytotoxic activity of saponins, especially those of ginseng and soy, was deeply investigated and reviewed [50,51,52]. These molecules are effective against different cancer cell lines, such as Hep-G2 (hepatocellular carcinoma cell line), HT1080 (fibrosarcoma cell line), HeLa (cervical cancer), HL-60 (promyelocytic leukemia cells) and MDA-MB-453 (breast cancer) [45]. A cytotoxic activity was demonstrated for different compounds, such as saxifragifolin B, saxifragifolin D [53], α-hederin [54], glochierioside A [55] and filiasparoside C [56].

Interestingly, this class of phytochemicals has been also investigated for its potential antidiabetic properties, with the aim to find new effective drugs in the treatment of diabetes mellitus. The hypoglycemic action of saponins seems to be due to different mechanisms of action, such as the restoration of the insulin response, the increase of plasma insulin levels and the induction of the release of insulin from the pancreas. For example, the saponin platyconic acid, isolated from Platycodi radix, was demonstrated to increase insulin-stimulated glucose uptake in 3T3-L1 adipocytes, whereas arjunolic acid, present in *Terminalia arjuna* Wight & Am. and other species, showed α-amylase and α-glucosidase inhibitory activity [57].

**Figure 1:**
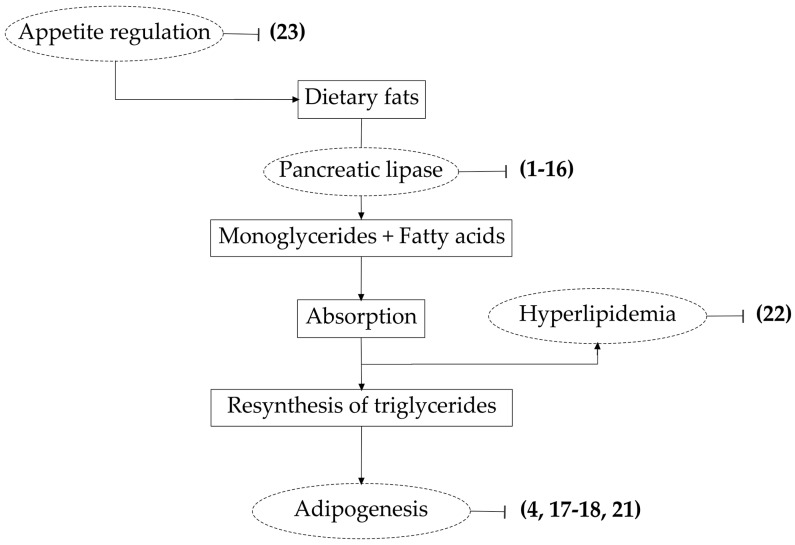
Schematic representation of the modes of action of saponins on lipid metabolism. Numbers between the brackets represent the molecules reported in Figure 2, Figure 3, Figure 4, Figure 5 and Figure 7.

The aim of the present review is to summarize the studies concerning the potential therapeutic efficacy of saponins against obesity. The obtained results about the various metabolites and active plant extracts are here grouped based on the different mechanisms of action observed (Figure 1).

## 4. Saponins and Pancreatic Lipase Inhibition

### 4.1. Lipase Inhibition

Lipases are enzymes responsible for fat digestion able to cleave long-chain dietary triglycerides into polar lipids. Lingual and gastric lipases are the first enzymes involved in fat digestion. They cleave short and medium chain triglycerides more efficiently than longer chain ones and cannot process sterols or phospholipids. Pancreatic lipase (PL) and other two lipolytic enzymes, carboxyl ester hydrolase and phospholipase A2, are secreted by the pancreas [58].

Pancreatic lipase is the most important human lipase and is associated with the hydrolysis of 50%–70% of total dietary fats. Lipase inhibition is one of the most important strategies advanced by pharmaceutical industries to decrease fat absorption after its ingestion. Orlistat (tetrahydrolipstatin, Xenical^®^) is a potent PL inhibitor and has been demonstrated to be effective in the treatment of obesity [8].

### 4.2. Lipase inhibition by Saponin-Containing Plant Extracts

Han and coworkers demonstrated the anti-obesity activity of the aqueous extract of *Platycodon grandiflorum* (Jacq.) A.DC. radix (Table 1). This extract was able to inhibit dietary fat absorption by inhibiting pancreatic lipase activity, and its ability to prevent obesity was also tested in vivo. Mice fed with a high-fat diet enriched with a 5% aqueous extract of *Platycodi radix* showed parametrial adipose tissue weights significantly lower than control-fed mice. Moreover, they were also characterized by a significantly lower plasmatic triacylglycerol concentration. It was supposed that the observed activity could be due to the total saponin fraction isolated from the aqueous extract. Interestingly, it was found that this fraction effectively inhibited pancreatic lipase activity in vitro [59].

Oishi and colleagues evaluated the anti-obesity potential of a saponin fraction from *Momordica charantia* L., discovering that it was able to inhibit the pancreatic lipase activity, as well as the elevation of the serum neutral fat level after corn oil loading in mice [60].

A pancreatic lipase inhibitory activity was also demonstrated by Hu and his colleagues for the hydroalcoholic extract of *Aesculus turbinata* Blume and its saponin fraction. The seeds of this plant contain a mixture of triterpenoidal saponins named escins. The anti-obesity potential of total escins was also evaluated in vivo, pointing out, in mice liver, a strong suppression of the increase in body weight, parametrial adipose tissue weight, hepatic triacylglycerol content and total cholesterol content [61].

Zheng and colleagues tested the activity of *Gypsophila oldhamiana* (Miq.). Interestingly, the water-soluble fraction obtained from the 95% EtOH extract of the plant roots, containing triterpenoid saponins, showed a strong inhibitory activity against pancreatic lipase (IC_50_ value of 0.54 mg/mL) [62].

### 4.3. Saponins Inducing Lipase Inhibition

**Figure 2:**
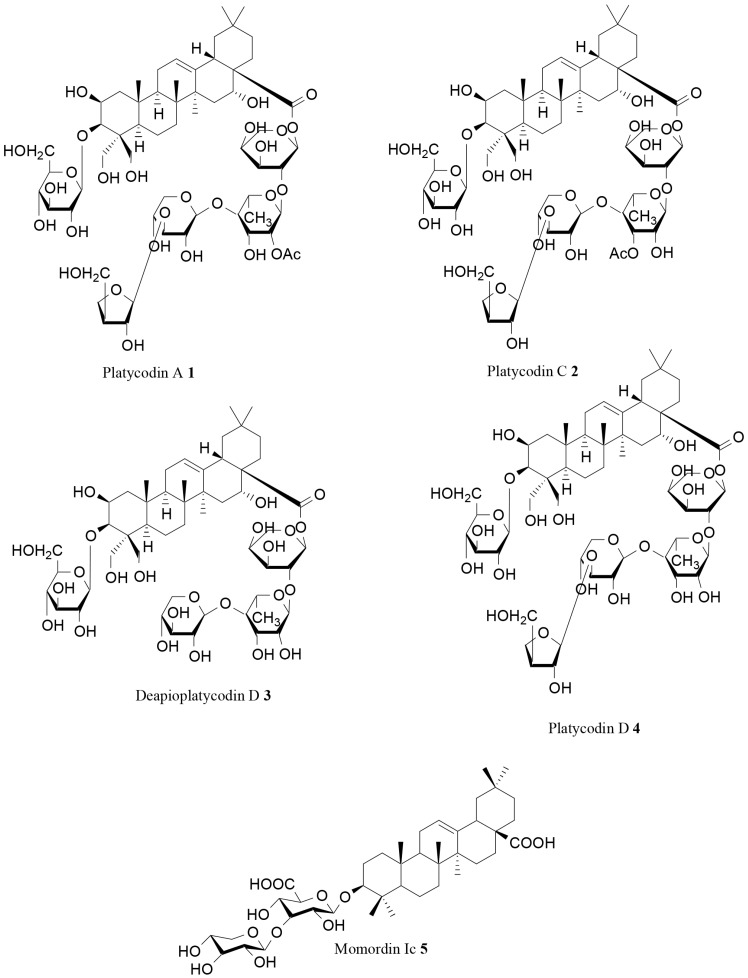
Structure of some saponins with inhibitory effects on pancreatic lipase [71,73].

An interesting pancreatic lipase inhibitory activity was demonstrated for some triterpenoidal saponins isolated from the roots of *Platycodon grandiflorum* (Jacq.) A.DC. [71], whose anti-obesity potential had been previously demonstrated by Han and coworkers [59]. Different known saponins were isolated and tested by Xu and coworkers. Among these molecules, a significant inhibitory effect on PL was demonstrated for platycodins A (**1**) and C (**2**; Table 2, Figure 2), with 3.3% and 5.2% pancreatic lipase activity vs. control, respectively, at a concentration of 500 µg/mL. A good activity was also observed for deapioplatycodin D (**3**) and platycodin D (**4**) (11.67% and 34.8% pancreatic lipase activity vs. control) [71].

The anti-obesity effect of platycodin saponins from Platycodi radix was also confirmed in vivo by Zhao and coworkers, which demonstrated their ability to induce body weight reduction in diet-induced obese rats [83].

Han and coworkers found that the ethanol extract of *Kochia scoparia* (L.) Schard. fruits was able to prevent the increases in body weight induced by the high-fat diet in mice. Moreover, they demonstrated that both raw extract and its saponin fraction were able to inhibit the elevation of the plasma triacylglycerol level after the oral administration of a lipid emulsion. The authors isolated and tested seven saponins from *K. scoparia* fruit. Some of these compounds, such as momordin Ic (**5**; Figure 1), were effective in inhibiting pancreatic lipase activity. According to these findings, the anti-obesity potential observed for *K. scoparia* was supposed to be linked to the effectiveness of isolated compounds on pancreatic lipase activity [73].

**Figure 3:**
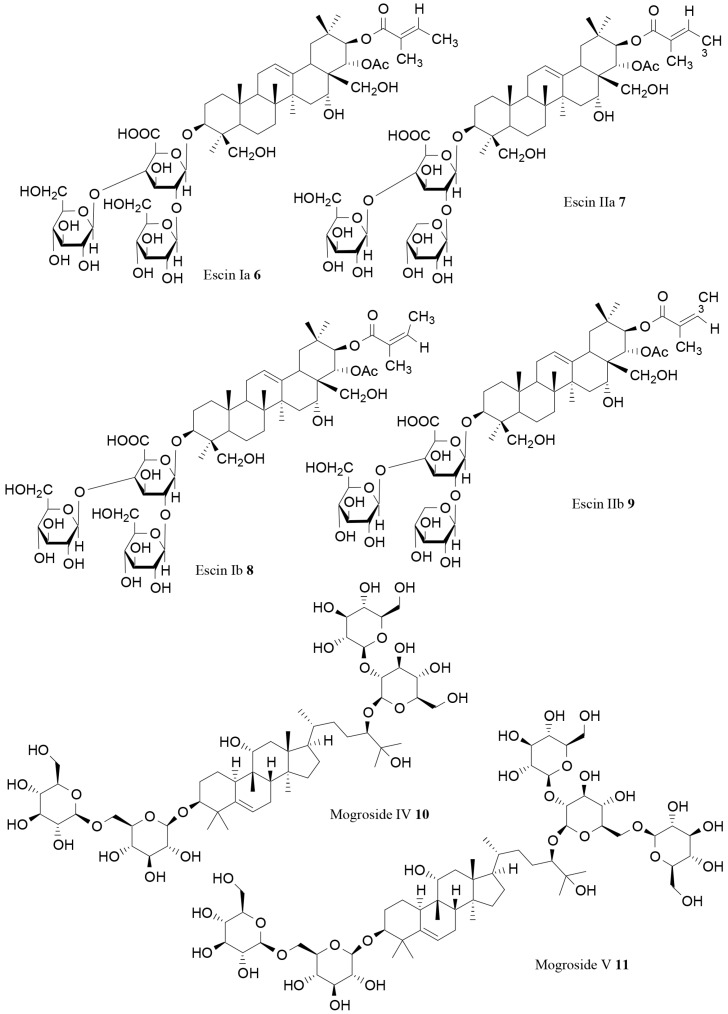
Other saponins able to inhibit pancreatic lipase [74,75].

A very interesting lipase inhibitory activity was observed for escins extracted from horse chestnut (*Aesculus turbinata* Blume). Kimura and coworkers examined the inhibitory effect of these triterpenoidal saponins. Escins Ia (**6**), IIa (**7**), Ib (**8**) and IIb (**9**) strongly inhibited pancreatic lipase activity, with IC_50_ values of 48, 61, 24 and 14 µg/mL, respectively (Figure 3). The derivatives deacetylescins and desacylescins were less active than escins [74].

A strong in vitro and in vivo anti-obesity activity was also observed for saponins extracted from *Siraitia grosvenorii* C. Jeffrey (Cucurbitaceae). The fruits of this plant contain a mixture of cucurbitane triterpene glycosides, named mogrosides, which were frequently used as an alternative to sugar for diabetic and obese patients due to their high sweetness. Sun and coworkers recently demonstrated the inhibitory effects of total mogrosides and mogrosides IV (**10**) and V (**11**; Figure 3) on pancreatic lipase in vitro (IC_50_ values of 517.73, 289.09 and 256.00 μg/mL, respectively). The in vivo effects of samples were also evaluated on male C57BL/6 mice fed a high-fat diet. Total mogrosides were able to suppress body weight increase, as well as abdominal and epididymal fats weight. Hepatic triacylglycerol and total cholesterol content in mice liver were also significantly affected [75].

**Figure 4:**
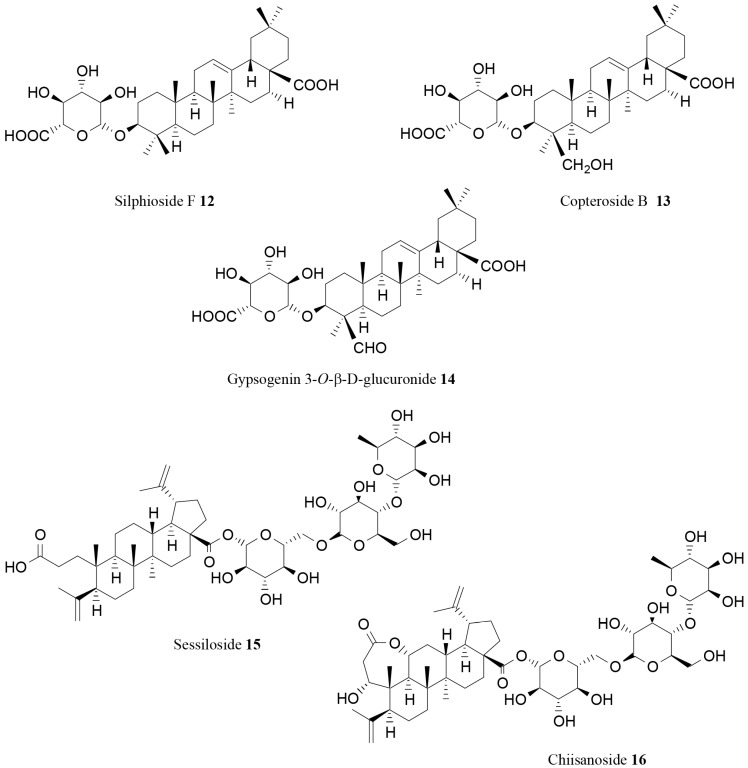
Further saponins inhibiting pancreatic lipase [76,77].

Some triterpenoid saponins inhibiting pancreatic lipase were also isolated from the fruits of *Acanthopanax senticosus* (Rupr. et Maxim.) Harms. Some of these molecules, such as silphioside F (**12**), copteroside B (**13**) and gypsogenin 3-*O*-β-d-glucuronide (**14**; Figure 4), showed a good inhibitory activity (IC_50_ values ranging from of 0.22–0.29 mM) [76].

An interesting activity on pancreatic lipase was also observed for the novel saponin sessiloside (**15**) and the known saponin chiisanoside (**16**) isolated from the leaves of *Acanthopanax sessiliflorus* (Rupr. et Maxim.) Seem. These two compounds inhibited lipase activity in a dose-dependent manner, with IC_50_ values equal to 0.36 and 0.75 mg/mL, respectively. Moreover, the administration of the saponin-rich fraction isolated from the extract of the plant was able to suppress body weight gain of mice fed a high-fat diet [77].

Gypenosides are other saponins that were found to inhibit porcine pancreatic lipase (PL) activity in a dose-dependent manner [84].

## 5. Saponins and Adipogenesis Inhibition

### 5.1. Adipogenesis

Excess energy is normally stored, through lipogenesis, in adipocyte cytoplasm in the form of triglycerides. However, severe obesity is characterized by an increased number of adipocytes through preadipocyte differentiation. The process by which undifferentiated preadipocytes are converted into fully-differentiated adipocytes is called adipogenesis, and it is a finely-regulated process involving concerted transcriptional and cellular events [79,81].

The control of adipogenesis through its many potential regulators could be a useful tool against obesity. For example, according to recent studies, the activation of AMP-activated protein kinase (AMPK) represents a potential strategy against obesity. AMPK is an important regulator of fat metabolism, which is able to control fatty acid synthesis and uptake, insulin secretion and glucose uptake in many tissues [80]. In particular, its activation stimulates β-oxidation and glucose uptake in skeletal muscle and inhibits hepatic fat and cholesterol synthesis. Because of these effects, this protein kinase appears to be an important emerging target for the treatment of metabolic syndrome, including not only obesity, but also type 2 diabetes [85].

Besides the role of AMPK, different adipogenic transcription factors, such as peroxisome proliferator-activated receptor γ (PPARγ), CCAAT/enhancer-binding protein alpha (C/EBPα) and sterol regulatory element-binding protein-1c (SREBP-1c), are key regulators of adipogenesis, as their suppression can inhibit preadipocyte differentiation [63].

### 5.2. Saponin Fraction and Pure Compounds Inhibiting Adipogenesis

**Figure 5:**
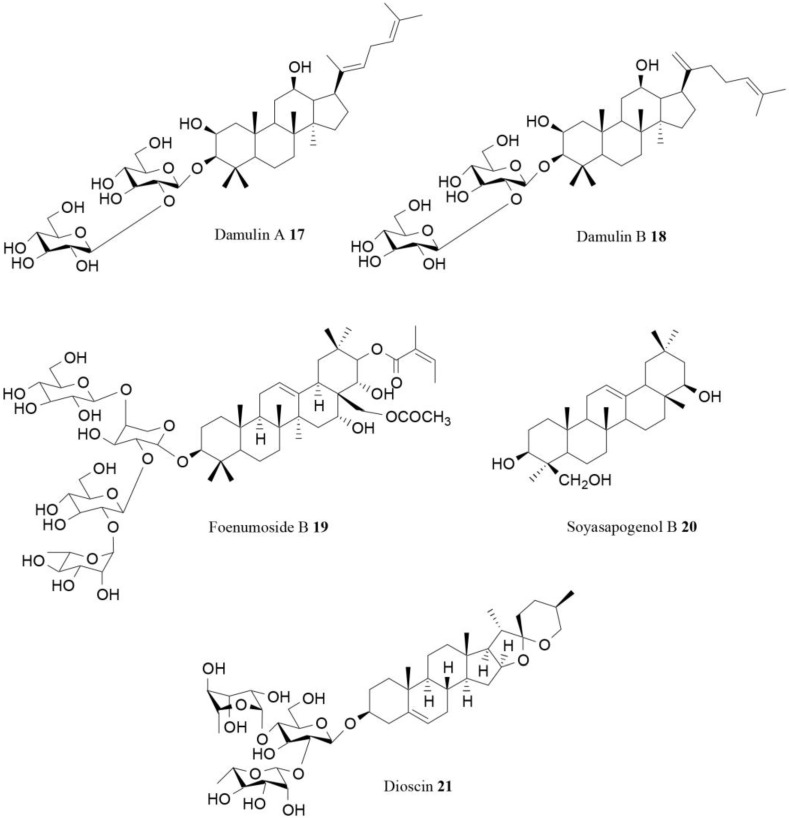
Saponins activating AMPK [78,79,80,81].

Two dammarane-type saponins with anti-obesity potential linked to AMPK regulation, damulin A (**17**; Figure 5) and damulin B (**18**), were isolated from *Gynostemma pentaphyllum* Makino (Cucurbitaceae). These two compounds were able to activate AMPK in cultured L6 myotubes [78].

The anti-obesity potential of this plant extract has been previously successfully evaluated by Megalli and coworkers. They demonstrated its ability to reduce triglyceride, total cholesterol and low density lipoprotein cholesterol levels in the obese Zucker fatty diabetic rat model [86]. Gauhar and coworkers evaluated the ability of the ethanol extract of *G. pentaphyllum* to activate AMPK. This extract was then subjected to physical modification by autoclaving at 121 °C for four hours. This process increased AMPK phosphorylation in L6 cells, and this result was correlated with elevated levels of AMPK activators, damulins A and B [85].

The triterpene saponin foenumoside B (**19**; Figure 5), isolated as an active component of *Lysimachia foenum-graecum* Hance, is another compound that inhibits adipocyte differentiation. The anti-adipogenic effect of foenumoside B was evaluated using preadipocyte 3T3-L1 cells that were differentiated into mature adipocytes in the presence of various concentrations of tested compound. Foenumoside B suppressed lipid accumulation with an IC_50_ value equal to 0.2 µg/mL. It was found that this molecule increased the phosphorylation of AMPK in a dose-dependent manner, suggesting a direct regulatory role on AMPK activation in adipocytes. Foenumoside B was also tested in vivo in a mouse model, and the oral administration was found to significantly reduce high-fat diet-induced body weight gain [79].

Hwang and coworkers verified the effects of the saponin fraction obtained from the aqueous extract of the roots of *Platycodon grandiflorum* (Changkil saponins) on AMP-activated protein kinase (AMPK) and hepatic lipogenesis in HepG2 cells. The obtained results indicated that saponins effectively stimulated AMPKα activation in HepG2 cells and inhibited lipid accumulation in HepG2 cells [10].

Furthermore, platycodin D (**4**; Figure 2) isolated from the root of *Platycodon grandiflorum* (Jacq.) A.DC., whose capability to inhibit pancreatic lipase has been already discussed [71], was tested for its capacity to inhibit adipogenesis. Lee and coworkers investigated its ability to decrease the expression of adipogenic factors through AMP-activated protein kinase α (AMPKα) in adipocytes. The ability to prevent abdominal fat accumulation in high-fat diet-induced obese C57BL/6 mice was also verified. In vitro results confirmed that platycodin D was able to reduce fat accumulation through the inhibition of adipogenic signal transcriptional factors, which function via AMPK signaling, such as CCAAT/enhancer binding protein α (C/EBPα) and peroxisome proliferator-activated receptor γ2 (PPARγ2) [72].

Kim and coworkers, interestingly, evaluated the anti-obesity effect of saponin compounds present in a traditional Korean fermented soy food named cheonggukjang, testing the capacity to affect triglycerides’ accumulation and AMPK activity in 3T3-L1 preadipocyte cells. Two groups of saponins were identified in cheonggukjang: soyasapogenols A and B. Triglycerides’ content in 3T3-L1 cells treated with the saponin extracts was significantly lower (17%–28%) than that in the control. Moreover, the saponins’ extract was able to increase AMPK activation. In particular, the saponin compound soyasapogenol B (**20**; Figure 5) effectively induced AMPK activation at the concentration of 2.5 μg/mL [80].

The steroidal saponins dioscin (**21**), which is present in several medicinal plants, was also demonstrated to be an effective inhibitor of adipogenesis. This molecule was able to suppress lipid accumulation in vitro in 3T3-L1 cells and its anti-adipogenic effect was linked to an influence on AMPK/MAPK during adipogenesis [81].

Yao and coworkers evaluated the influence of quinoa (*Chenopodium quinoa* Willd.) saponins on the differentiation of 3T3-L1 preadipocytes. The fraction isolated from this plant was able to inhibit triglyceride accumulation in the mature adipocytes. The authors demonstrated that quinoa saponins significantly down-regulated the mRNA and protein expression of key adipogenic transcription factors PPARγ and C/EBPα and also reduced mRNA and protein expression of sterol SREBP-1c [63].

## 6. Saponins and Appetite Regulation

### 6.1. Control of Food Intake and Energy Homeostasis

An important role in the regulation of body weight is played by the central nervous system (CNS), which receives numerous neural impulses from the gastrointestinal mucosa and fat tissue and controls food intake and energy expenditure (thermogenesis) [87,88].

The involved neurons modulate the hypothalamic–pituitary–adrenal axis. The gut peptides signaling to the hypothalamus mediate the appetite-stimulating effect through the activation of neurons containing neuropeptide Y (NPY) and agouti-related peptide (AgRP) or, on the contrary, an appetite-inhibitory effect via other neurons. NPY constitutes an important regulator: it increases food intake and reduces dietary fat oxidation [89,90]. The role of many peptides, such as NPY, AgRP, cholecystokinin (CCK), ghrelin and glucagon-like peptide 1 (GLP-1), has been taken into account. Leptin and insulin are also involved in hypothalamic appetite regulation, and they constitute potential therapeutic targets to treat obesity [89].

Leptin is an adipocyte-derived protein that acts as a regulator of energy homeostasis. This regulator acts centrally, inhibiting the synthesis of NPY [91].

### 6.2. Saponins Affecting the Expression of Appetite Peptides

Kim and coworkers investigated the in vivo anti-obesity effects of the crude saponin fraction of Korean red ginseng (*Panax ginseng* C.A. Meyer). Both rats fed a high-fat diet and control rats fed a normal diet were treated with this fraction, and body weight and food consumption were monitored. The authors also investigated the expression of appetite peptides, such as leptin and NPY. The saponin fraction was demonstrated to reduce body weight by 20%–30% and food intake in both normal and high-fat diet rats. Moreover, the treatment reduced the expression of hypothalamic NPY and serum leptin in high-fat diet rats [64].

**Figure 6:**
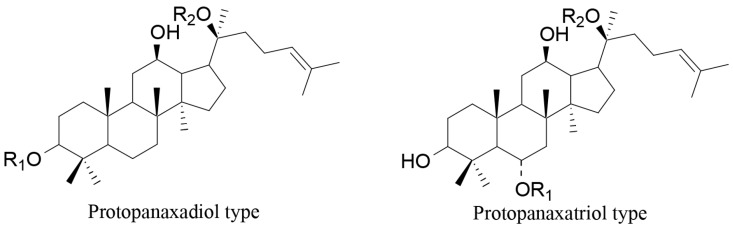
Protopanaxadiol and protopanaxatriol type saponins [89,92].

A further study dealt with the evaluation of the in vivo anti-obesity activity of the two major active compounds isolated from crude saponins of red ginseng: protopanaxadiol and protopanaxatriol type (Figure 6) [89,92]. The first group was more active, suggesting that protopanaxadiol type saponins are the principal phytochemicals responsible for the anti-obesity activity of ginseng saponins. However, both saponin types were able to reduce body weight, food intake and leptin in the high-fat diet group rats. It was observed that, after both treatments, the hypothalamic expression of orexigenic neuropeptide Y was significantly decreased, whereas the anorexigenic cholecystokinin was increased compared with the control high-fat diet group [89].

The flower buds of *Camellia sinensis* L. were also demonstrated to have anti-obesity effects through the suppression of the appetite signals in the hypothalamus. Hamao and coworkers observed that the methanolic extract of this plant was able to inhibit body weight gain in high-fat diet-fed mice and to suppress liver weight, liver triglyceride and the weight of visceral fat. Food intake was also inhibited in a dose-dependent manner. In particular, the *n*-butanol soluble fraction of *C. sinensis* was able to inhibit food intake at a dose of 250 mg/kg. It was demonstrated that this fraction significantly suppressed mRNA levels of neuropeptide Y [66].

## 7. Further Effect of Saponins on Lipid Metabolism: Anti-Hyperlipidemic Activity

### 7.1. Hyperlipidemia

Hyperlipidemia has been defined as elevated cholesterol and triglycerides levels in plasma, and it represents one of the major risk factors associated with coronary heart disease [93]. The incidence of hyperlipidemia has increased worldwide because of an augmented fat consumption [84]. Statins and fibrates are some effective available hypolipidemic drugs. However, the use of synthetic drugs may cause some adverse effects, such as nausea, diarrhea, myositis, gastric irritation and hyperuricemia. Therefore, as previously observed for obesity, the interest of the researchers is towards new natural products with hypolipidemic properties and with minimal or no side effects [82,94].

### 7.2. Saponin-Rich Extracts and Pure Compounds with Antihyperlipidemic Activity

**Figure 7:**
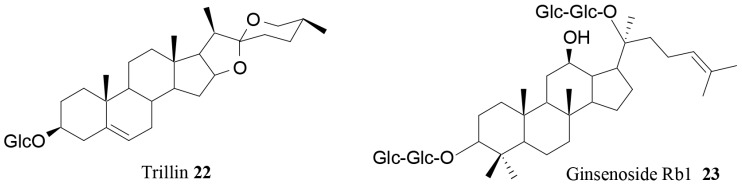
Further saponins with anti-obesity properties [65,95].

Trillin (**22**; Figure 7), a steroidal saponin isolated from *Dioscorea nipponica* Makino rhizome, showed a strong anti-hyperlipidemic activity. The *n*-butanol fraction obtained from the ethanol extract of the plant was able to reduce the high-fat diet-induced upregulation of cholesterol and triglyceride in rats and to restore HDL and LDL to the normal status. Different beneficial effects were exerted by the intra-peritoneal administration of trillin: bleeding and blood coagulation time were significantly improved, and the levels of cholesterol, LDL and HDL were restored back to the normal conditions. Moreover, this saponin improved the levels of lipid peroxidation and superoxide dismutase activity [82].

Elekofehinti and coworkers evaluated the antihyperlipidemic activity of the saponin fraction from *Solanum anguivi* Lam. fruits. The effects were evaluated in alloxan-induced diabetes rats. The administration of saponins (20–100 mg/kg for 21 days) significantly reduced the elevated glucose levels, total cholesterol, total triglycerides and LDL compared to the diabetic control, while HDL levels were increased [67].

A significant hypolipidemic effect was also observed for *Achyranthes aspera* L. seeds. The saponin fraction isolated from this plant was tested on the serum lipid profile of albino rats fed a high cholesterol diet. The sample was administered for four weeks inducing a significant decrease of total cholesterol, total triglycerides and LDL and a significant increase of HDL level [68].

## 8. Other Saponin Containing-Fractions Affecting Weight Reduction

*Panax ginseng* CA Meyer, a species already discussed, is known for the elevated content of bioactive saponins. Karu and coworkers assayed the effects of the saponin fraction extracted from the roots of the plant on male Balb/c mice. Ginseng saponins were able to inhibit the increase in body weight and decrease the hypertriacylglycerolemia induced by a high-fat diet. The same authors also tested the ability to inhibit pancreatic lipase in vitro, demonstrating a dose-dependent inhibition [65].

Crude saponins from stems and leaves of *Panax quinquefolium* L. showed in vivo anti-obesity activity, as well [69]. The ginsenoside fraction was administered (1 g/kg body weight) to female ICR mice fed a high-fat diet for eight weeks. The treatment decreased parametrial adipose tissue weight and inhibited the elevations of plasma triacylglycerol. Furthermore, crude saponins were also demonstrated to inhibit pancreatic lipase in vitro. In a further study, Liu and coworkers tested the effects of the two types of ginsenosides isolated from the leaves of *P. quinquefolius* L.: protopanaxadiol and protopanaxatriol types [96]. The first group was effective, inhibiting pancreatic lipase activity in a dose-dependent manner, while protopanaxatriol saponins showed no activity. The anti-obesity effect of the active saponin fraction was then tested in vivo in mice fed a high-fat diet, and the obtained results demonstrated that it was able to reduce the adipose tissue weights and serum and liver triglycerides level compared to the control high-fat diet group.

An interesting anti-obesity potential was demonstrated for *Ilex paraguariensis* A. St. Hilaire (mate) [97]. Mate preparations were traditionally considered to be appetite stimulators [95], but more recent studies have instead demonstrated the effectiveness in weight management [97,98,99,100,101]. More recently, de Resende and colleagues evaluated the effectiveness of a purified saponin fraction extracted from *I. paraguariensis* [95]. This fraction was significantly more effective in reducing fat weight and glucose oxidation of hepatic and adipose tissue in healthy rats fed a standard diet than the whole extracts obtained from mate leaves and unripe fruits and induced also a significant lowering of the blood triglycerides level in rats. Thus, the observed in vivo activities could be ascribed to the mate saponin fraction.

Latha and coworkers assessed the anti-obesity potential of *Achyranthes aspera* L. saponin-rich extract on male Wistar rats fed a high-fat diet [102]. A dose of 120 mg/kg induced a significant reduction of food intake, body weight and visceral organ weights. Moreover, serum levels of total cholesterol, triglycerides, very low density lipoproteins (VLDL) and low density lipoproteins (LDL) were reduced, while the levels of high density lipoproteins (HDL) were increased compared to the high-fat diet control group.

As already observed for flower buds of *Camellia sinensis* L. [66], an anti-obesity activity was also demonstrated for the fruit peel extract of this plant. Chaudhary and coworkers observed that the administration of this extract (100 mg/kg/day) significantly decreased body weight in rats fed high-fat diet [103].

An in vivo anti-obesity activity was also observed for the saponin fraction isolated from *Gymnema sylvestre* R. Br. aqueous leaf extract [70]. The sample was administered to high-fat diet-induced obese rats at a dose of 100 mg/kg body weight for eight weeks. Food consumption and body weight were significantly decreased, as well as visceral organs’ weight and triglycerides, total cholesterol, low-density lipoproteins and very low-density lipoproteins.

Lin and colleagues tested the in vivo anti-obesity potential of ginsenoside Rb1 (**23**, Figure 7), the major component of ginseng [104]. The sample was injected intraperitoneally in mice at the dose of 20 mg/kg for three weeks, and both decreased weight gain and food intake were observed compared to the control high-fat group. Moreover, Rb1 caused the reduction of blood glucose and some lipids, and it was demonstrated to modulate serum levels of peptide YY (PYY) and NPY.

A significant in vivo anti-obesity potential was also observed for Chikusetsu saponins isolated from rhizomes of *Panax japonicus* C.A. Meyer [105]. This saponin fraction was able to prevent the increases in body weight and parametrial adipose tissue weight induced by a high-fat diet and inhibited the elevation of the plasma triacylglycerol level in female ICR mice. Moreover, the inhibition of pancreatic lipase activity was also demonstrated in vitro.

## 9. Conclusions

Obesity is a chronic disease of increasing prevalence worldwide closely associated with hyperlipidemia, diabetes, hypertension and cardiovascular diseases. The pathogenesis of obesity is very complex, and it involves different factors, such as dietary habits, genetic predisposition and metabolism.

Weight control programs often include diet control, physical activity and drugs. The effectiveness of medicinal plants as natural supplements to reduce body weight has been taken into account with the aim to find new remedies with better efficacy and lower adverse effects compared to drugs used until now. Different secondary metabolites from plants have been demonstrated to modulate body weight. Besides their known biological activities, saponins have been recently investigated for their anti-obesity potential. Emerging evidence suggests that these compounds can have beneficial effects against obesity through different mechanisms of action. Both saponin-rich extracts and pure compounds have been demonstrated, for instance, to inhibit pancreatic lipase or to modulate adipogenesis and appetite.

However, at present, there is not sufficient evidence able to support the clinical application of saponins in the treatment of obesity. Future clinical trials on the safety and effectiveness of these compounds are needed to validate the effects of saponins observed in vitro and in animal models.

## Figures and Tables

**Table 1:** Saponin-containing plant extracts with anti-obesity activity.

Plant Species	Plant Part	Study	Action	Reference
*Platycodon grandiflorum* (Jacq.) A.DC.	Roots	In vitro In vivo	Lipase inhibition	[10,59]
*Momordica charantia* L.	_	In vitro In vivo	Lipase inhibition	[60]
*Aesculus turbinata* Blume	Seeds	In vitro In vivo	Lipase inhibition, suppression of body weight increase, hepatic triacylglycerol content and total cholesterol content	[61]
*Gypsophila oldhamiana* (Miq.)	Root	In vitro	Lipase inhibition	[62]
*Chenopodium quinoa* Willd.	Seeds	In vitro	Downregulation of adipogenic transcription factors	[63]
*Panax ginseng* C.A. Meyer	Roots	In vitro In vivo	Lipase inhibition, downregulation of hypothalamic NPY and serum leptin	[64,65]
*Camellia sinensis* L	Flower buds	In vivo	Suppression of mRNA levels of neuropeptide Y	[66]
*Solanum anguivi* Lam.	Fruits	In vivo	Antihyperlipidemic activity	[67]
*Achyranthes aspera* L.	Seeds	In vivo	Antihyperlipidemic activity	[68]
*Panax quinquefolium* L.	Leaves	In vitro In vivo	Lipase inhibition, decrease of adipose tissue weight	[69]
*Gymnema sylvestre* R. Br.	Leaves	In vivo	Decrease of food consumption and body weight	[70]

**Table 2:** Saponins with anti-obesity activity.

Saponin	Plant Species	Study	Action	Reference
Platycodin A (**1**)	*Platycodon grandiflorum* (Jacq.) A.DC.	In vitro	Lipase inhibition	[71]
Platycodin C (**2**)	*Platycodon grandiflorum* (Jacq.) A.DC.	In vitro	Lipase inhibition	[71]
Deapioplatycodin D (**3**)	*Platycodon grandiflorum* (Jacq.) A.DC.	In vitro	Lipase inhibition	[71]
Platycodin D (**4**)	*Platycodon grandiflorum* (Jacq.) A.DC.	In vitro In vivo	Lipase inhibition, AMPK activation, prevention of abdominal fat accumulation	[71,72]
Momordin Ic (**5**)	*Kochia scoparia* (L.) Schard	In vitro	Lipase inhibition	[73]
Escin Ia (**6**)	*Aesculus turbinata* Blume	In vitro	Lipase inhibition	[74]
Escin IIa (**7**)	*Aesculus turbinata* Blume	In vitro	Lipase inhibition	[74]
Escin Ib (**8**)	*Aesculus turbinata* Blume	In vitro	Lipase inhibition	[74]
Escin IIb (**9**)	*Aesculus turbinata* Blume	In vitro	Lipase inhibition	[74]
Mogroside IV(**10**)	*Siraitia grosvenorii* C. Jeffrey	In vitro	Lipase inhibition	[75]
Mogroside V (**11**)	*Siraitia grosvenorii* C. Jeffrey	In vitro	Lipase inhibition	[75]
Silphioside F (**12**)	*Acanthopanax senticosus* (Rupr. et Maxim.) Harms	In vitro	Lipase inhibition	[76]
Copteroside B (**13**)	*Acanthopanax senticosus* (Rupr. et Maxim.) Harms	In vitro	Lipase inhibition	[76]
Gypsogenin 3-*O*-β-d-glucuronide (**14**)	*Acanthopanax senticosus* (Rupr. et Maxim.) Harms	In vitro	Lipase inhibition	[76]
Sessiloside (**15**)	*Acanthopanax sessiliflorus* (Rupr. et Maxim.) Seem	In vitro	Lipase inhibition	[77]
Chiisanoside (**16**)	*Acanthopanax sessiliflorus* (Rupr. et Maxim.) Seem	In vitro	Lipase inhibition	[77]
Damulin A (**17**)	*Gynostemma pentaphyllum* Makino	In vitro	AMPK activation	[78]
Damulin (**18**)	*Gynostemma pentaphyllum* Makino	In vitro	AMPK activation	[78]
Foenumoside B (**19**)	*Lysimachia foenum-graecum* Hance	In vitro In vivo	AMPK activation, reduction of body weight gain	[79]
Soyasapogenol B (**20**)	Korean fermented soy food named cheonggukjang	In vitro	AMPK activation	[80]
Dioscin (**21**)	Several species	In vitro	Influence on AMPK/MAPK	[81]
Trillin (**22**)	*Dioscorea nipponica* Makino	In vivo	Antihyperlipidemic activity	[82]
Ginsenoside Rb1 (**23**)	Ginseng	In vivo	Modulation of serum levels of PYY and NPY	[67]

## Abbreviations

The following abbreviations are used in this manuscript:

AgRP	agouti-related peptide
AMPK	AMP-activated protein kinase
C/EBPα	CCAAT/enhancer-binding protein alpha
CB1	cannabinoid type 1 receptors
CCK	cholecystokinin
CNS	central nervous system
EGCG	epigallocatechin gallate
EMEA	European Agency for the Evaluation of Medicinal Products
FDA	Food and Drug Administration
GLP-1	glucagon-like peptide 1
HDL	high density lipoproteins
HeLa	human cervical cancer cells
Hep-G2	hepatocellular carcinoma cell line
HL-60	promyelocytic leukemia cells
HT1080	fibrosarcoma cell line
ICD	International Classification of Diseases
LDL	low density lipoproteins
MAPK	mitogen-activated protein kinase
MDA-MB-453	breast cancer cell line
NPY	neuropeptide Y
PL	pancreatic lipase
PPARγ	peroxisome proliferator-activated receptor γ
PYY	peptide YY
SREBP-1c	sterol regulatory element-binding protein-1c
VLDL	very low density lipoproteins
WHO	World Health Organization
